# Effects of Minimalist vs. Traditional Running Shoes on Abdominal Lumbopelvic Muscle Activity in Women Running at Different Speeds: A Randomized Cross-Over Clinical Trial

**DOI:** 10.3390/s24020310

**Published:** 2024-01-05

**Authors:** María García-Arrabé, Marta de la Plaza San Frutos, Alberto Bermejo-Franco, Rebeca del Prado-Álvarez, Javier López-Ruiz, José Angel del-Blanco-Muñiz, María-José Giménez

**Affiliations:** 1 Faculty of Sport Sciences, Universidad Europea de Madrid, Tajo s/n, 28670 Villaviciosa de Odón, Spain; maria.gararrabe@universidadeuropea.es (M.G.-A.); marta.delaplaza@universidadeuropea.es (M.d.l.P.S.F.); alberto.bermejo@universidadeuropea.es (A.B.-F.); rebeca.delprado@universidadeuropea.es (R.d.P.-Á.); javier.lopez3@universidadeuropea.es (J.L.-R.); joseangel.delblanco@universidadeuropea.es (J.A.d.-B.-M.); 2 Department of Radiology, Rehabilitation and Physiotherapy, Complutense University of Madrid, Avda. Complutense s/n, 28040 Madrid, Spain

**Keywords:** CORE, electromyography, minimalist shoes, runners, running

## Abstract

This study aimed to investigate if the characteristics of different running shoes could influence intra-abdominal pressure during running. A single-centre, randomized, prospective cross-over clinical trial was performed measuring activity patterns of internal oblique (IO), lumbar erector (LE), and gluteus maximus (GM) muscles in healthy women when running with minimalist shoes (MS). Participants were randomly allocated into two-sequence (MS/TS or TS/MS) treadmill running at six, nine, and eleven km/h. The surface electromyographic activity of IO, LE, and GM muscles were recorded while running. A repeated measures ANOVA explored the interaction effects of three-muscle x three speeds x two shoes. Significance was set at *p* ≤ 0.05. Fifty-one healthy nulliparous women (mean age: 26.55 ± 5.11 years; body mass index: 21.29 ± 2.07 Kg/m^2^) were included. Our findings revealed lower activations of the LE compared to the internal oblique IO and GM, irrespective of running speed and footwear used. Electromyographic activation significantly increased with higher running speeds (*p* < 0.001) for all muscles, regardless of the type of footwear. Although electromyographic records with MS consistently showed higher values than those with TS, the differences were not statistically significant for all muscles at all speeds. Our results indicate that electromyographic activation patterns vary according to the muscle group, exhibiting higher values with increased running speed. No significant differences were observed between MS and TS.

## 1. Introduction

In recent years, the number of runners in the entire population has increased, with a special rise in the number of female participants in popular competitions, both in short-distance races and in half-marathons and marathons [1]. Despite the numerous benefits of running for improving quality of life, injuries are frequent, and 68.3% of runners reported having had an injury in the last year [2].

During a race, body tissues (muscles, fascia, tendons, bones, etc.) are stressed by different forces, mainly tension, compression, and torsion. Injuries occur when these impacts are greater than the absorption threshold of the tissues; therefore, the control of load peaks generated by these forces is essential for the prevention and even the treatment of pathologies due to overload [3]. One of the fastest ways to modulate loading rates is through the footwear that athletes wear [4]. However, despite the great technological development of footwear since the 1970s, injury rates have not decreased [2]. In the last decade, this has led to the emergence of minimalist shoes (MS) as a footwear alternative [5]. MS are those shoes that minimally intervene in the natural movement of the foot due to their high flexibility, low drop, low weight, thin sole thickness, and absence of movement and stability control devices, as defined in the Delphi study conducted by Esculier et al. [6].

Several studies have compared kinematic, biomechanical and electromyographic activity changes occurring in the lower limb with traditional shoes (TS) vs. MS, and have concluded that changes in cushioning do alter the loading rate of the impact force [7,8].

Running with MS has been promoted to reduce the risk of injury; a published article described that those runners in TS are almost three times more likely to report foot and ankle injuries than those in MS, but the type of injury was not specified [7]. Lieberman et al. [9] indicated that whereas TS promote rearfoot strike, thus increasing impact peaks, MS facilitate forefoot strike, softening the impact. In this sense, several studies have supported that runners commonly having a forefoot strike have fewer musculoskeletal injuries diagnosed in the lower limb [10,11,12].

However, to our knowledge, there are no studies investigating how the different characteristics of running shoes affect the abdomino-lumbopelvic region, despite it being well known that running implies a repeated and abrupt increase in intra-abdominal pressure, and can lead to the development of different dysfunctions such as stress urinary incontinence, chronic lower back pain, pubalgia, etc. [13,14].

The internal oblique (IO) and the erector lumbar (LE) muscles (anterior and posterior limits of the abdominal-lumbar-pelvic region, respectively), and the gluteus maximus (GM) are muscles involved in the control of abdominal pressure, with a key stabilizing role during the race. Knowledge of their behavior when running at different speeds, with different footwear, would help to identify shoes better at absorbing the impact derived from the race, thus better protecting the abdomino-lumbopelvic region. Using electromyography (EMG), a technique developed to measure the electrical activity of muscles, previous studies have shown variations in movements with respect to the relationship between footwear and running gait [15].

EMG is able to detect imbalances, alterations in muscle coordination or muscular fatigue [16], and has been used to investigate activity in other regions and muscles such as the peroneal, biceps femoris, gastrocnemius, and tibialis anterior muscles [17,18].

The hypothesis of this study was that discernible variations in the activity patterns of IO, LE, and GM muscles can be observed in healthy women when running with minimalist footwear as opposed to traditional footwear. The primary objective of this study was to explore the short-term effects of running with minimalist shoes versus traditional shoes on the activity patterns of IO, GM, and LE muscles in healthy women at different speeds (6, 9, and 11 km/h).

## 2. Materials and Methods

### 2.1. Study Design

A single-center, analyst-blinded, randomized, cross-over clinical trial was performed in the School of Nursing, Physiotherapy and Podiatry of the Complutense University of Madrid (Spain) from December 2020 to March 2021. Healthy female university students, nulliparous, between 18 and 38 years old, with a BMI < 30 kg/m^2^, who regularly used TS in sports practice, participated in the study. The sample was captured by publicizing the study in different university centers in Madrid (Complutense University and the European University) and on social networks for a period of 3 months (December 2020–February 2021). Exclusion criteria were professional athletes; pregnancy; presence of red flags at the lumbopelvic level (tumor, cauda equina syndrome, infectious process in the cervical spine, spinal cord compression derived from fracture, and abdominal aneurysm); kinesiophobia; urogynecological dysfunction; and lower limb surgeries in the last 6 months.

The local Research and Ethics Committee of the Hospital Clinical San Carlos (Madrid, Spain) approved the study protocol (code 19/570-E_TFM). The study was conducted in accordance with all the principles set forth in the Declaration of Helsinki, and all participants signed informed written consent forms to participate. The study was registered in the clinical trials data base (ClinicalTrials.gov; CI NCT04457141; accessed on 31 May 2022).

Voluntary subjects attended the study centre on Day 0 to assess eligibility criteria and to collect demographic data of eligible women until the required sample size was reached. Afterwards, subjects were randomized into two groups (MS/TS or TS/MS), according to the order of use of footwear: MS or TS running shoes. Each participant was assigned to one of the study groups using sealed envelopes with the sequence to follow. Study codes were used for the identification of participants, thus blinding the analyst for subsequent analysis of the electromyographic records.

### 2.2. Type of Footwear

**Figure 1:**
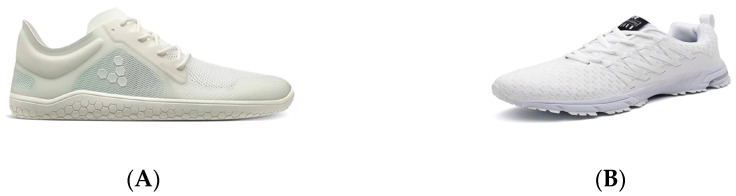
Minimalist (**A**) and traditional (**B**) footwear used in the study.

Two types of running shoes were used for the intervention. The MS used were Vivobarefoot Primus Lite III (Vivobarefoot Ltd., London, UK) with a minimalist index of 84%, 181 g weight, 10 mm heel thickness, drop 0, great longitudinal and torsional flexibility, without technological devices. The TS used were Sollomensi (Sollomensi, Beijing, China), exhibiting the following average characteristics of shod: 34% MI, 214 g weight, 10 mm heel thickness, drop 20 mm, great longitudinal and torsional resistance to deformation, with four technological devices in the midsole, calcaneal dome, insole and widening of the sole. Figure 1 shows the MS and TS used in the study.

### 2.3. Intervention Protocol

The intervention started with a 5 min warm-up at the pace chosen by the participant followed by three 30 s bouts of running at 6 km/h, 9 km/h and 11 km/h each. A wash-out period of 5 min in which participants remained seated and changed footwear was then followed by an identical above-described series of running with the new footwear. All subjects ran on the same treadmill (HP Cosmos treadmill, Mercury model; Ref.cos 30000va08, Hp/cosmos sport & medical, Nussdorf-Traunstein, Germany). All interventions were carried out under the supervision of the researcher/physiotherapist which assured compliance with the study procedures.

Electromyographic activity was measured during running at different speeds using TS and MS running footwear. The maximum and minimum peaks, and the total average of activity of IO, LE and GM muscles were recorded. To this end, the validated surface EMG mDurance^®^ (mDurance Solutions SL, Granada, Spain) [19], a portable sEMG system consisting of three parts: sensors, mobile computing and data analysis in the cloud, was used. Two bipolar sensors (Shimmer sensor, Shimmer Research Ltd., Dublin, Ireland) were used for collection of superficial muscle activity. The mDurance^®^ mobile application installed in a Galaxy A7 Android Tablet (ZtotopCase, Suwon, Republic of Korea) recorded data received from the Shimmer unit and transferred it to a cloud service where it was stored, filtered, and sEMG signals analyzed to generate reports.

Electromyographic activity was recorded unilaterally (right side) from the IO, LE and GM muscles through surface electrodes. The electrodes used were pregelled Ag/AgCl (Ref. 019-400400, Natus Medical Incorporated, Middleton, WI, USA) with a diameter of 10 mm and a distance between electrodes of 20 mm. Two bipolar sensors were used for the acquisition of surface muscle activity, ( Shimmer sensor, Shimmer Research Ltd., Dublin, Ireland). The first sensor (MDUR-4B1A) had wires connected to electrodes placed on the IO. In addition, an electrode was placed on the ASIS as a reference electrode. The sensor was then strapped to the front of the leg. The second sensor (MDUR-4B05) was fitted with electrode cables to record the activity of the EL and GM. An electrode was also placed on the sacrum as a reference electrode. The sensor was then strapped to the dorsal spine.

**Figure 2:**
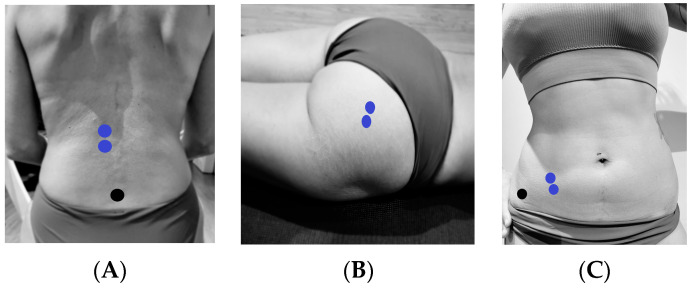
(**A**) Location of electrodes (blue) of erector lumbar (LE) and reference electrodes (black). (**B**) Location of electrodes (blue) of gluteus maximus (GM). (**C**) Location of electrodes (blue) of internal oblique (IO) and reference electrodes (black).

To monitor the electromyographic activity of the LE muscle, two contiguous electrodes were placed in the craniocaudal direction with 3 cm between them, lateral to the spinous process of L1 [20], with another placed as a reference electrode on the anterior superior iliac spine. For the electromyographic activity of the IO muscle, two contiguous electrodes were placed in the craniocaudal direction in the triangle formed by the inguinal ligament, the anterior superior iliac spine (ASIS), and the umbilical midline [20]. For the GM muscle, two electrodes were placed 2 cm apart lateral to the median sacral crest in the craniocaudal direction [21] (Figure 2).

### 2.4. EMG Data Analysis

Before carrying out the running protocol, electromyographic activity was recorded in the standing resting position during a 2 s standing resting position, wherein participants maintained a static, relaxed posture prior to the commencement of the running protocol. This served as a baseline or reference point for subsequent electromyographic (EMG) analyses, allowing us to capture the resting state of the muscles prior to dynamic activity.

**Figure 3:**
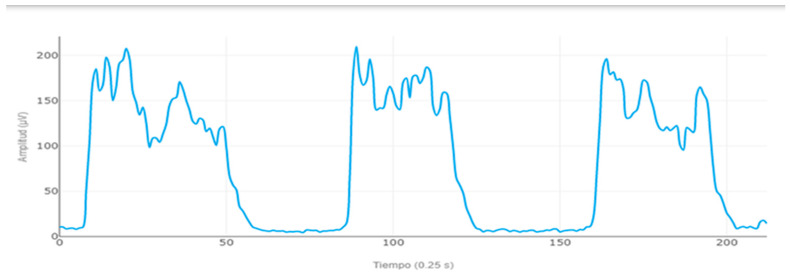
Electromyography of erector spinae to assess MVIC with MDurance^®^.

The maximum voluntary isometric contraction (MVIC) for each muscle was also electromyographically recorded by performing three isometric contractions of 10 s, with 20 s of rest between each repetition. For the IO MVIC assessment, subjects were placed in a supine position on the table with the lower limbs flexed and were asked to perform an abdominal crunch with homolateral rotation. For the GM MVIC assessment, participants were placed in a prone position on the table with the right leg flexed, and they were asked to perform a hip extension while raising the thigh on the table. For the LE MVIC assessment, participants were placed in a prone position on the table with the lower legs flexed, executing trunk extension (Figure 3).

**Figure 4:**
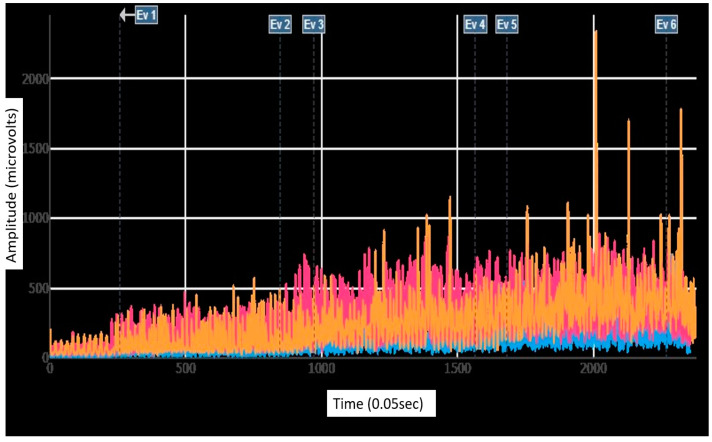
EMG activation of erector lumbar (LE) (blue), gluteus maximus (GM) (pink), and internal oblique (IO) (orange) muscles during running at three different speeds, (between Ev1–Ev2: 6 km/h), (between Ev3–Ev4: 9 km/h), (between Ev5–Ev6: 11 km/h).

To enhance the accuracy of subsequent electromyographic analyses during each participant’s treadmill race, signals were subjected to meticulous filtering procedures. A high-pass filter with a frequency cutoff of 20 Hz was employed to eliminate low-frequency noise and interference, while a low-pass filter with a frequency cutoff of 450 Hz was applied to attenuate high-frequency noise. These filtering parameters were carefully selected to capture the relevant frequencies associated with muscle activity during a dynamic task. Following this, the electromyographic signals were normalized using the MVIC values monitored at the beginning of the session. Subsequently, a detailed analysis of the normalized electromyographic data was conducted, determining the total average, average minimum peaks, and average maximum peaks for each running speed (6, 9, and 11 km/h) (Figure 4). This multi-step approach ensured robust data processing and provided a comprehensive understanding of muscular demands during treadmill running.

The rectification of the electromyographic signal was performed using mDurance software version 1, implementing absolute rectification to transform the original signal into a unipolar representation. This transformation was achieved by taking the absolute value of each point in the signal.

The calculation of the “average of maximum peaks” was computed by averaging the first 10 peak values within each electromyographic analysis period, encompassing 3 different speeds of 6, 9, and 11 km/h. Similarly, we applied the same approach to the “average of minimum peaks”.

Normalization of the rectified signal was carried out by calculating the root mean square (RMS) over a specific interval. Subsequently, we divided the rectified signal by the RMS to express the amplitude in relative terms.

### 2.5. Statistical Analyses

**Figure 5:**
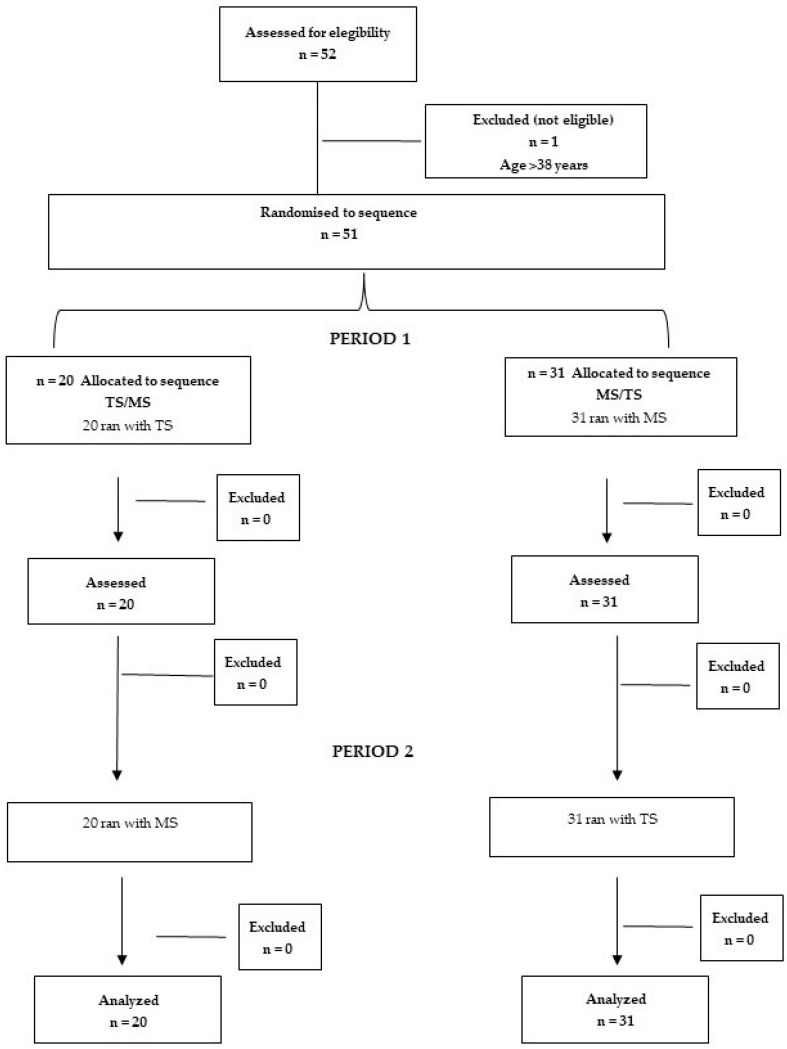
Flow diagram of the study.

The convenience sample size was based on a previous study assessing the abdominal muscle strength in nulliparous female athletes, which included a total of 44 participants [22]. Considering a potential 15% loss of data due to electromyographic crosstalk, the required sample size was finally set at 52 participants. Figure 5 shows the flow diagram of the study.

SPSS 25.0 software (IBM SPSS Statistics, IBM, New York, NY, USA) was used for data analysis and JAMOVI 2.0 software for data analysis and figure creation. Kolmogorov–Smirnov’s test was used for the normality assumption. The sphericity of the data was evaluated with the Mauchly test. The distribution of the three electromyographic activity variables (maximum, minimum and total average peaks) showed a great variability with high asymmetry values. Due to this, a logarithmic transformation of data was performed, and analyses were carried out with the transformed values. A repeated measures analysis of variance was performed analyzing the three-muscle (OI, GM, LE) × three speeds (6, 9 and 11 km/h) × two shoes (MS and TS) design, exploring main and interaction effects. The Bonferroni correction post hoc analysis was used. The level of significance was set at *p* ≤ 0.05 and confidence intervals at 95%.

## 3. Results

A total of 51 women were included in the study: 20 in Group TS/MS and 31 in Group MS/TS. All participants completed the study. Table 1 shows the demographic data of study participants.

Table 2 shows median electromyographic activation (maximum and minimum peaks, and total average) of the three muscles measured at the different running speeds.

**Figure 6:**
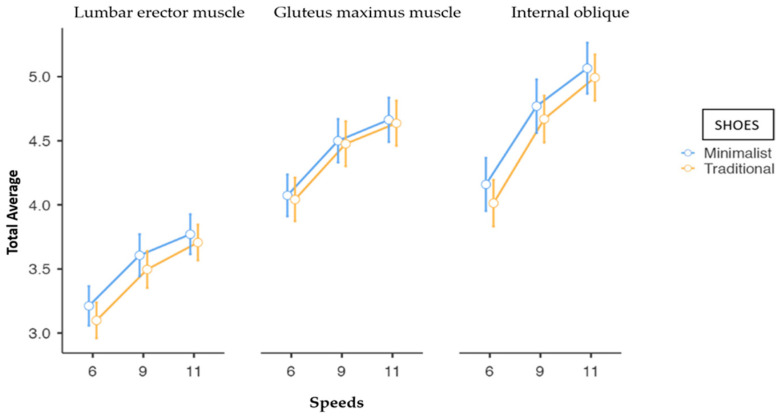
Estimated marginal mean of electromyographic activity scores by speed and by shoes in erector lumbar (LE), gluteus maximus (GM), and internal oblique (IO). Error bars represent standard error of the mean.

Regardless of the speed and the footwear used, the activation of the LE muscle was always lower than activations of GM and IO muscles. For all muscles, with both footwear (analyzed separately), a statistically significant (*p* < 0.001) increase in the electromyographic activation was obtained by increasing the speed. Greater changes were observed between 6 km/h and 9 km/h than between 9 km/h and 11 km/h (Figure 6).

Table 3 shows differences and pairwise comparisons of electromyographic activation between shoes (MS/TS) by speed. Although the EMG records with MS were higher than those with TS, differences were small and non-statistically significant for all muscles at all speeds.

## 4. Discussion

The aim of the present study was to compare the short-time effects of running at different velocities with TS vs. MS on three muscles (IO, GM, and LE) of the abdomino-lumbopelvic region, in nulliparous women. To this end, electromyographic activities in the muscles were registered and three variables (total average, minimum peaks, and maximum peaks) were analyzed. The results showed that electromyographic activation varied depending on the muscle; the LE showed the lowest activation during the race when compared with the GM and IO muscles. In all cases, the activation increased when increasing the speed, but differences between MS and TS running shoes were not statistically significant despite muscle activations always being greater with MS than with TS.

The IO and GM muscles are, due to their location, usually covered by thick layers of adipose tissue which could have increased their EMG signals [23,24], in contrast to the LE muscle where surface electrodes were placed on skin regions immediately above the muscle, minimizing possible interferences. This might be a plausible explanation for the lower electromyographic signal found in the LE muscle in the present study. In this sense, it has also been stated that the coverage of muscles by different fascial tissues and superficial aponeurosis affects the signal amplitude in surface EMG, causing differences in the EMG signal for each muscle [24]. In the case of the IO muscle, the proximity of other muscles such as the anterior rectus abdominis, the external oblique, or the transversus abdominis could be the cause of the phenomenon called “crosstalk” (interference) caused by signals from neighboring muscles, as has been reported [25].

Despite an earlier study showing different activation of the distal musculature in lower limbs (tibialis anterior, gastrocnemius and soleus) with TS vs. MS [26], in the present study we were not able to detect differences in the abdomino-lumbopelvic muscles considered. In the absence of published data on this body region, our results could only be compared to those of a previous study reporting an increase in the electromyographic activity in the GM and gluteus medius in the flight phase, and a pre-activation of the foot support prior to landing [26]. The fact that in the present study, the phases of the race were not fragmented, and the muscular activity registered at each speed during 30 s. was average without considering the different stages of the race, could be the basis for the differences.

Fast running leads to a greater recruitment of motor units, a muscular adaptation to the increased production of force necessary when running at higher speeds [27], and also to a possible increase in synchronization between the active motor units [25]. Lieberman et al. [28] described an increased GM activation linked to increases in running speed and uphill running. These are two activities associated with the forefoot landing typical of the MS-generated pattern. This argument worked in favor of our hypothesis, the higher activation with MS (forefoot strike) than with TS; however, in this study, higher activation was found when increasing speed with both shoes but not when comparing the different footwear.

Another possible explanation for the absence of differences observed when comparing racing with each type of shoe could be due to the short period of the monitored running. Different authors [29] have reported that having prior experience in the use of MS was a “sine qua non” condition for detecting changes in the tread pattern, indicating that in the first attempts of running barefoot, a complete transition to a changed pattern is not guaranteed. The possibility that the immediate changes produced by the different running shoes impacted the organization of the entire lower limb, (as detailed in other studies [30,31]), but not more proximal areas such as the abdomino-lumbo-pelvic region, could not be discarded and warrants further investigation.

This study has several limitations. All participants were novice MS runners, they were not used to running on a treadmill, and the speed was externally predetermined (not chosen by themselves); all these facts could have altered the normal biomechanical and muscle activation patterns. In addition, the duration of the race was 90 s, a short time in which the runner may not reach the same expected muscle fatigue as in real situations such as longer races or with the presence of uneven surfaces. In this sense, the association of fatigue with alterations in the pattern activity has been described [25]. The high variability typical of EMG [32] also represents a limitation despite following the SENIAM (surface electromyography for the non- invasive assessment of muscle) international protocol [33]. Wide ranges of values in the measured parameters derived from the high variability of EMG could have blurred statistical differences between TS and MS running.

On the other hand, to our knowledge this is the first study investigating the short-time impact of different running shoes on muscles of the abdomino-lumbopelvic region. Despite differences between shoes not being detected, a study finding was the description of higher muscular activation of body proximal muscles when increasing running speeds, as previously described for distal muscles. Another strength to highlight is the cross-over study design that allowed pairwise comparisons, strengthening EMG data through minimizing its high variability.

## 5. Conclusions

The electromyographic activation exhibited variations dependent on the muscle, with higher activations observed in the GM and IO muscles compared to the LE in nulliparous women during running. Additionally, an increase in muscle activation was noted with the escalation of running speed. Notably, no statistically significant differences were found in the electromyographic activity of the three muscles when comparing the use of traditional shoes (TS) versus minimalist shoes (MS).

Considering the practical implications, these findings suggest that the choice of footwear may not significantly affect electromyographic activity in the muscles of the abdomino-pelvic region evaluated during running in nulliparous women. However, it is important to note that the observed increase in muscle activation with higher race speeds emphasizes the importance of considering running speed in training programs targeting the abdomino-lumbopelvic region.

For future research, we propose studies that examine long-term changes in electromyographic activity in the abdomino-lumbopelvic muscles associated with a progressive transition to minimalist footwear. Additionally, incorporating 3D biomechanical pattern monitoring alongside electromyographic activity can provide a more comprehensive understanding of the effects of minimalist footwear on the function of these muscles during running. The inclusion of metrics such as median frequency will also be considered in future research to enrich the analysis of neuromuscular response to changes in footwear during physical activity.

## Figures and Tables

**Table 1:** Demographic characteristics of study participants (n = 51).

Variable	Mean ± SD	Range
Age (years)	26.55 ± 5.11	(20.0–38.0)
Weight (kg)	58.24 ± 7.06	(45.0–82.0)
Height (m)	1.65 ± 0.06	(1.53–1.82)
BMI ^1^ (Kg/m^2^)	21.29 ± 2.07	(17.28–27.40)

^1^ BMI: Body Mass Index.

**Table 2:** Electromyographic activity: maximum peaks, minimum peaks, and total average (μV) of the three muscles measured at different running speeds when running with both footwear types.

		Running Speed					
Variable	Muscle	6 km/h		9 km/h		11 km/h	
		MS ^a^	TS ^b^	MS ^a^	TS ^b^	MS ^a^	TS ^b^
Maximum peaks	LE ^c^	48.9	51.6	74.0	73.5	89.8	96.21
		(33.7, 112.0)	(32.0, 106.7)	(50.7, 156.5)	(43.5, 155.8)	(55.5, 181.2)	(51.1, 179.1)
	GM ^d^	163.0	193.5	232.8	238.4	254.1	278.2
		(65.8, 354.4)	(63.5, 373.8)	(87.0, 551.5)	(88.2, 553.9)	(104.8, 633.7)	(104.0, 605.0)
	IO ^e^	174.5	190.6	360.3	403.0	543.3	442.6
		(59.3, 545.7)	(62.9, 449.1)	(115.9, 860.1)	(115.4, 738.4)	(139.7, 1150.1)	(156.6, 972.0)
Minimum peaks	LE ^c^	4.8	3.9	7.7	7.6	9.25	7.95
		(2.6, 8.2)	(2.6, 8.9)	(4.0, 15.0)	(3.7, 15.0)	(4.2, 18.7)	(4.3, 17.3)
	GM ^d^	10.8	12.8	19.1	19.6	21.3	24.0
		(4.7, 25.0)	(3.7, 24.0)	(8.0, 53.0)	(5.5, 45.7)	(9.1, 59.7)	(7.6, 60.2)
	IO ^e^	14.5	13.8	29.3	20.4	32.9	30.0
		(5.8, 31.4)	(5.4, 21.0)	(10.23, 58.1)	(10.2, 47.8)	(14.3, 84.0)	(14.5, 77.2)
Total average	LE ^c^	17.9	18.0	26.0	29.7	36.0	35.1
		(12.6, 38.2)	(11.0, 37.6)	(17.9, 53.0)	(15.0, 56.8)	(19.8, 63.1)	(18.4, 67.7)
	GM ^d^	60.3	54.9	86.3	78.7	95.0	96.6
		(23.7, 120.0)	(20.3, 119.2)	(36.9, 187.2)	(32.8, 219.6)	(46.5, 233.1)	(40.6, 218.5)
	IO ^e^	61.2	63.6	141.4	136.4	186.1	157.9
		(19.7, 187.9)	(23.7, 135.1)	(38.3, 319.6)	(45.2, 253.0)	(56.0, 378.2)	(60.1, 378.8)

^a^ MS: minimalist shoes; ^b^ TS: traditional shoes; ^c^ LE: lumbar erector muscle; ^d^ GM: gluteus maximus muscle; ^e^ IO: internal oblique muscle. Data are shown as median (Q1, Q3).

**Table 3:** Pairwise comparison of electromyographic activation when running with both types of footwear by speed.

				Running Speed			
		6 km/h		9 km/h		11 km/h	
Variable	Muscle	Difference (95% CI)	*p*	Difference (95% CI)	*p*	Difference (95% CI)	*p*
Maximum peaks	LE ^a^	0.12 (−0.13, 0.37)	0.33	0.11 (−0.12, 0.33)	0.35	0.07 (−0.17, 0.29)	0.55
	GM ^b^	0.06 (−0.10, 0.22)	0.47	0.0 (−0.17, 0.17)	0.99	0.02 (−0.14, 0.18)	0.82
	IO ^c^	0.10 (−0.19, 0.39)	0.50	0.10 (−0.16, 0.36)	0.44	0.09 (−0.16, 0.33)	0.47
Minimum peaks	LE ^a^	0.11 (−0.05, 0.27)	0.17	0.10 (−0.09, 0.29)	0.28	0.08 (−0.10, 0.26)	0.38
	GM ^b^	0.04 (−0.13, 0.21)	0.66	0.04 (−0.15, 0.23)	0.67	0.04 (−0.14, 0.22)	0.66
	IO ^c^	0.21 (−0.03, 0.45)	0.08	0.18 (−0.05, 0.40)	0.12	0.10 (−0.13, 0.32)	0.39
Total average	LE ^a^	0.11 (−0.08, 0.31)	0.25	0.11 (−0.10, 0.32)	0.29	0.06 (−0.12, 0.25)	0.49
	GM ^b^	0.03 (−0.12, 0.19)	0.68	0.02 (−0.15, 0.19)	0.78	0.03 (−0.13, 0.19)	0.74
	IO ^c^	0.15 (−0.12, 0.41)	0.27	0.10 (−0.15, 0.35)	0.43	0.07 (−0.16, 0.31)	0.54

^a^ LE: lumbar erector muscle; ^b^ GM: gluteus maximus muscle; ^c^ IO: internal oblique muscle. Data are shown as difference of activation (minimalist/traditional) (95% confidence interval).

## Data Availability

Data are contained within the article.
